# Structure-Antiplatelet Activity Relationships of Novel Ruthenium (II) Complexes: Investigation of Its Molecular Targets

**DOI:** 10.3390/molecules23020477

**Published:** 2018-02-22

**Authors:** Chih-Hsuan Hsia, Thanasekaran Jayakumar, Joen-Rong Sheu, Shin-Yi Tsao, Marappan Velusamy, Chih-Wei Hsia, Duen-Suey Chou, Chao-Chien Chang, Chi-Li Chung, Themmila Khamrang, Kao-Chang Lin

**Affiliations:** 1 Graduate Institute of Medical Sciences and Department of Pharmacology, College of Medicine, Taipei Medical University, Taipei 110, Taiwan; d119102013@tmu.edu.tw (C.-H.H.); tjaya_2002@yahoo.co.in (T.J.); sheujr@tmu.edu.tw (J.-R.S.); d119106003@tmu.edu.tw (C.-W.H.); fird@tmu.edu.tw (D.-S.C.); 2 Division of Endocrinology & Metabolism, Department of Internal Medicine, Sijhih Cathay General Hospital, New Taipei 22174, Taiwan; metaendocr@yahoo.com.tw; 3 Department of Chemistry, North Eastern Hill University, Shillong 793022, India; mvelusamy@gmail.com; 4 Department of Cardiology, Cathay General Hospital, Taipei 106, Taiwan; change@seed.net.tw; 5 Division of Pulmonary Medicine, Department of Internal Medicine, Taipei Medical University Hospital, Taipei 106, Taiwan; clchung@tmu.edu.tw; 6 Department of Neurology, Chi Mei Medical Center, Tainan 710, Taiwan

**Keywords:** ruthenium complexes, platelets, ATP, [Ca^2+^]i, Akt-JNK-p38, Lyn-Fyn-Syk, SAR

## Abstract

The regulation of platelet function by pharmacological agents that modulate platelet signaling has proven to be a positive approach to the prevention of thrombosis. Ruthenium complexes are fascinating for the development of new drugs, as they possess numerous chemical and biological properties. The present study aims to evaluate the structure-activity relationship (SAR) of newly synthesized ruthenium (II) complexes, TQ-1, TQ-2 and TQ-3 in agonists-induced washed human platelets. Silica gel column chromatography, aggregometry, immunoblotting, NMR, and X-ray analyses were performed in this study. Of the three tested compounds, TQ-3 showed a concentration (1–5 μM) dependent inhibitory effect on platelet aggregation induced by collagen (1 μg/mL) and thrombin (0.01 U/mL) in washed human platelets; however, TQ-1 and TQ-2 had no response even at 250 μM of collagen and thrombin-induced aggregation. TQ-3 was effective with inhibiting collagen-induced ATP release, calcium mobilization ([Ca^2+^]i) and P-selectin expression without cytotoxicity. Moreover, TQ-3 significantly abolished collagen-induced Lyn-Fyn-Syk, Akt-JNK and p38 mitogen-activated protein kinases (p38 MAPKs) phosphorylation. The compound TQ-3 containing an electron donating amino group with two phenyl groups of the quinoline core could be accounted for by its hydrophobicity and this nature might be the reason for the noted antiplatelet effects of TQ-3. The present results provide a molecular basis for the inhibition by TQ-3 in collagen-induced platelet aggregation, through the suppression of multiple machineries of the signaling pathway. These results may suggest that TQ-3 can be considered a potential agent for the treatment of vascular diseases.

## 1. Introduction

Cardiovascular diseases (CVDs) are the number one killer and thrombosis in particular is accountable for the majority of CVD-related mortalities [1]. Platelets play crucial roles in hemostasis by preventing excessive blood loss during vascular damage through blood clotting. Nevertheless, abnormal activation of platelets leads to thrombosis in pathological conditions of rupturing atherosclerotic plaques [2]. Thrombosis decreases the blood supply to the heart and brain resulting in heart attacks and strokes. Therefore, targeting platelets has been confirmed to be operative in the prevention and treatment of CVDs [3,4]. Despite the presently used anti-platelets aspirin and clopidogrel exhibiting worthwhile results in many patients, they cause adverse side effects such as bleeding complications [5]. Thus, the progress of potential therapeutic strategies for the prevention and treatment of thrombotic diseases demands further study.

Organometallic complexes have been considered for therapeutic applications in various pathological conditions [5,6]. For instance, cisplatin—a platinum-based Food and Drug Administration (FDA)-approved anti-cancer drug—increased the survival rate of patients with testicular cancer [7] and has been used to treat different types of cancer [8]. The achievement of cisplatin and its dose-limiting side effects inspired important investigation in this area. Ruthenium-based organometallics are an encouraging class of therapeutic compound, as two ruthenium agents, NAMI-A and KP1019, have entered human clinical trials [9,10]. Experimental studies established that numerous ruthenium-based compounds display high cytotoxicity towards a wide range of cancer cells with reduced side effects [11]. It has been specified that three specific properties make ruthenium an interesting metal for drug development: (i) its range of oxidation states; (ii) its ability to mimic iron binding under physiological conditions and (iii) its low toxicity compared to platinum [12]. Our recent study established that a novel ruthenium-based compound TQ-5 suppressed platelet aggregation in vitro in washed human platelets via inhibiting the phosphorylation of Akt and JNK1 and subsequently reducing the ATP release reaction and intracellular calcium mobilization [13]. Another interesting study from our group also showed that the ruthenium compound TQ-6 has a novel role in inhibiting platelet activation through the inhibition of the agonist receptors-mediated inside-out signaling such as Src-Syk-PLCγ2 cascade and subsequent suppression of granule secretion, leading to disturbing integrin α_IIb_β_3_-mediated outside-in signaling and ultimately inhibiting platelet aggregation [14]. In this study, the structure activity relationship (SAR) of three newly synthesized ruthenium-based compounds—TQ-1, TQ-2 and TQ-3—was performed in collagen and thrombin-induced washed human platelet aggregation. The possible molecular target was also examined.

## 2. Results 

### 2.1. Molecular Structure of Complexes

**Figure 1:**
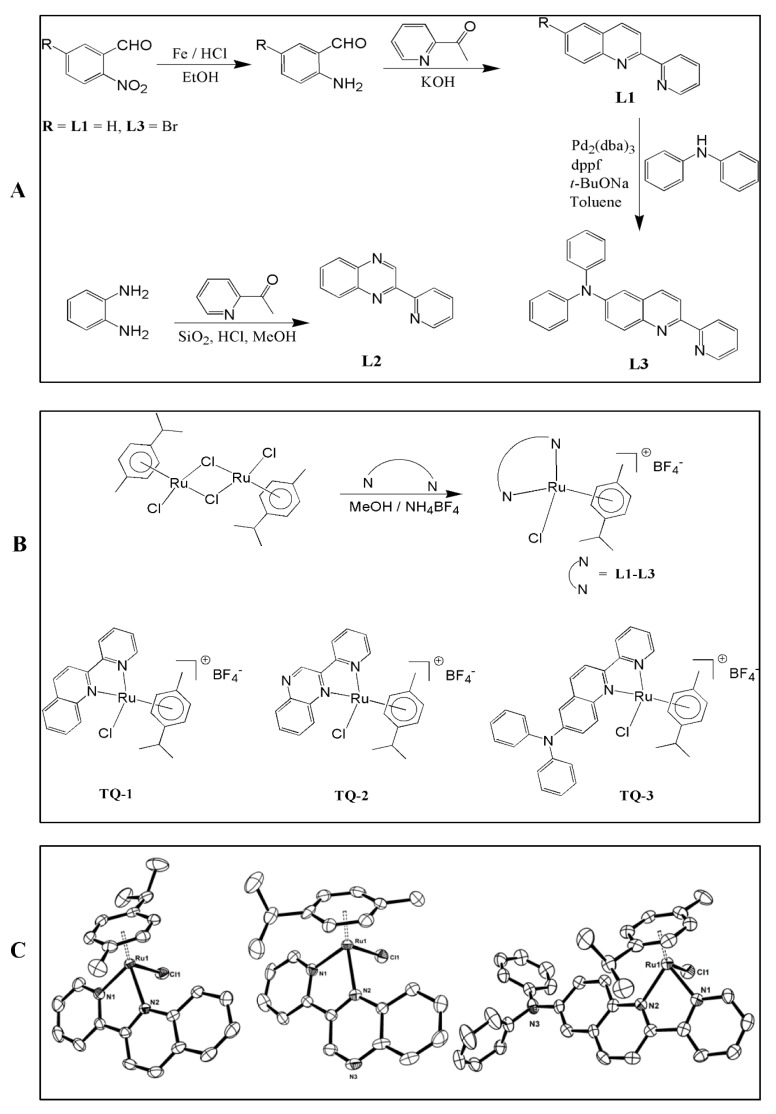
Scheme of synthesis of ligands 2-pyridin-2-yl-quinoline (L1) and 2-pyridin-2-yl-quinoxaline (L2) and diphenyl-(2-pyridin-2-yl-quinolin-6-yl)-amine (L3) (**A**); Scheme of synthesis of complexes [Ru(η^6^-cymene)(L1)Cl]BF_4_ (TQ-1), [Ru(η^6^-cymene)(L2)Cl]BF_4_ (TQ-2) and [Ru(η^6^-cymene)(L3)Cl]BF_4_ (TQ-3) (**B**); ORTEP drawings of TQ-1, TQ-2 and TQ-3. Hydrogen atoms and BF_4_ anion are omitted for clarity (**C**).

The molecular structures of some of the complexes were investigated by single-crystal X-ray studies. The crystallographic details and structure refinement details are summarized in Table S1. The geometrical parameters around the metal atom involving ring centroid are listed in Table S2. The Oak Ridge Thermal-Ellipsoid Plot (ORTEP) program views of the complexes are shown in Figure 1B. The ruthenium complex TQ-1 crystallized in the monoclinic system with P21/n space group. Complexes TQ-2 and TQ-3 are crystalized in the triclinic system with P-1 space group. The coordination geometry around Ru(II) in the complexes is best described as pseudo-octahedral with the arene ring occupying the three coordination sites in a η^6^-fashion and the two nitrogen atoms of the bidentate ligands and a chloride ion occupying the remaining coordination sites. The Ru-N bond lengths fall in the range 2.072–2.109 Å (TQ-1: 2.072, 2.108; TQ-2: 2.081, 2.109; TQ-3: 2.078, 2.109 Å) and the Ru-Cl bond length in the range 2.390–2.40 Å (TQ-1: 2.398, TQ-2: 2.40, TQ-3: 2.390 Å) [15]. The six Ru–C bonds have almost comparable bond distances with an average Ru–C bond lengths of 2.202 (TQ-1); 2.212 (TQ-2); 2.193 (TQ-3) Å and the ruthenium-arene centroid ring distance (Ru-Ct, 1.684 Å) is almost the same for all the complexes and is similar to those in related Ru(II)-arene complexes [16].

### 2.2. Electronic Spectra

UV-Vis spectra of the ligands and complexes were recorded in acetonitrile and the resulting data (Figures S1 and S2) are summarized in the experimental section. In the absorption spectra, all the ligands display a band located in the range 335–396 nm, which possibly originates from the π → π* transitions located in the chromophores such as quinoline and quinoxaline moieties [17]. The incorporation of diphenyl amine on the quinoline ring of 2-pyridin-2-yl-quinoline (L1) to obtain diphenyl-(2-pyridin-2-yl-quinolin-6-yl)-amine (L3) leads to an additional band at 396 nm, which is expected of the electron releasing amino group enhance the electron density on the quinoline ring. The intense absorption bands were noticed in the ranges of 223–321 nm and were assigned to intra ligand n→π*, π→π* transitions. The UV-Vis spectra of all complexes exhibit intense absorption bands in the range 240–370 nm, assigned to intraligand π–π* transitions. The lowest-energy absorption in each case (421–465 nm) is tentatively assigned as metal-to-ligand charge transfer (MLCT) transition from the 4d orbitals of Ru(II) to the empty π* ligand orbitals [18]. Further, the MLCT transition of the complexes is following the order of 465 (TQ-3) > 445 (TQ-2) > 421 (TQ-1) nm. The incorporation of amino group on the quinoline ring of TQ-1 to TQ-3 and additional nitrogen atom (TQ-2) causing red-shift in the band position illustrating that the change in electronic nature of ligand by additional nitrogen atoms enhances the electron density on the aromatic rings.

### 2.3. Influence of TQ-1, 2 and 3 on Collagen and Thrombin-Induced Platelet Aggregation

**Figure 2:**
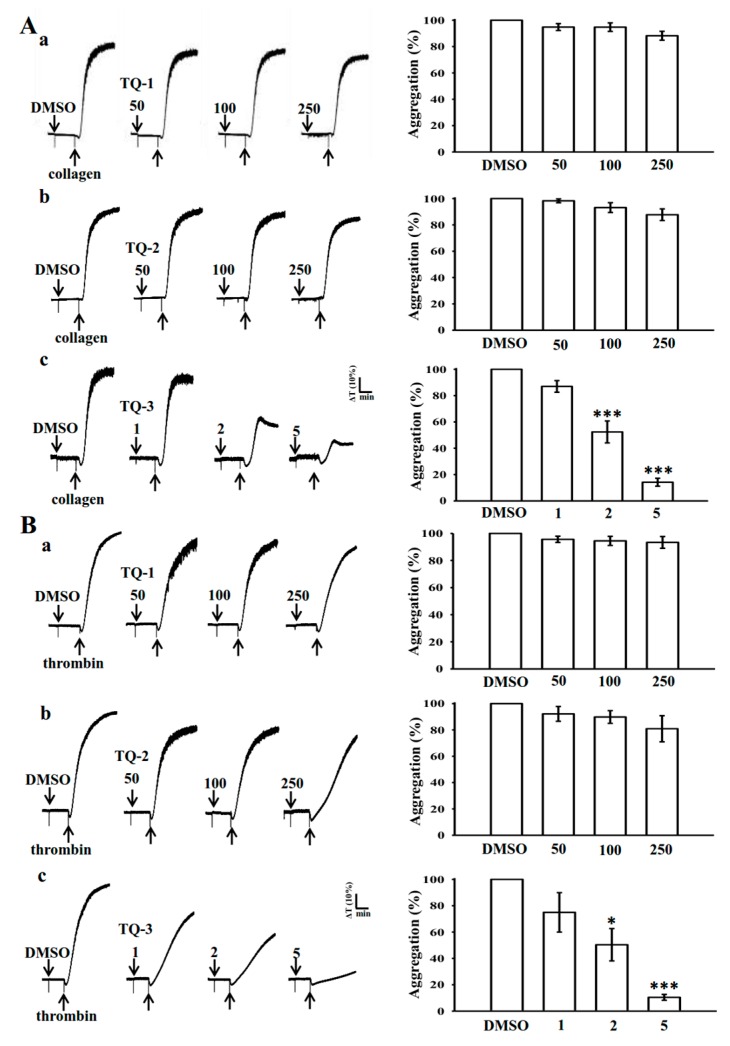
Inhibitory activity of ruthenium complexes (TQ-1-TQ3) on (**A**) collagen and (**B**) thrombin–induced platelet aggregation in washed human platelets. Washed human platelets (3.6 × 10^8^ cells/mL) were pre-incubated with the solvent control (0.1% DMSO) or TQ-1, TQ-2 (50–250 μM) and TQ-3 (1–5 μM) and then treated with 1 μg/mL collagen and 0.01 U/mL thrombin to stimulate platelet aggregation. Data are presented as means ± standard errors of the means (*n* = 4). * *p* < 0.05, *** *p* < 0.001 compared with the DMSO group.

The schematic diagram for the synthetic procedure of ligands, complex and ORTEP has been given in Figure 1. The concentration of collagen and thrombin induced maximal human platelet aggregation, that was approximately 1 µg/mL and 0.01 U/mL, respectively, thus, these concentrations were used in this study. Platelets were stimulated with collagen and thrombin to analyze the effect of TQ-1, TQ-2 and TQ-3 on washed human platelet aggregation. Off these three compounds, TQ-3 concentration dependently (1–5 µM) inhibited the signal intensities, which we derived from aggregometer in collagen (Figure 2A,c) and thrombin (Figure 2B,c)-induced platelet aggregation. Nevertheless, TQ-1 and TQ-2 did not alter the signal on either collagen (Figure 2A,a,b) or thrombin (Figure 2B,a,b)-induced aggregation at any tested concentrations.

### 2.4. Effect of TQ-1, 2 and 3 on Cytotoxicity, [Ca^2+^]i Mobilization and ATP Release in Human Platelets 

**Figure 3:**
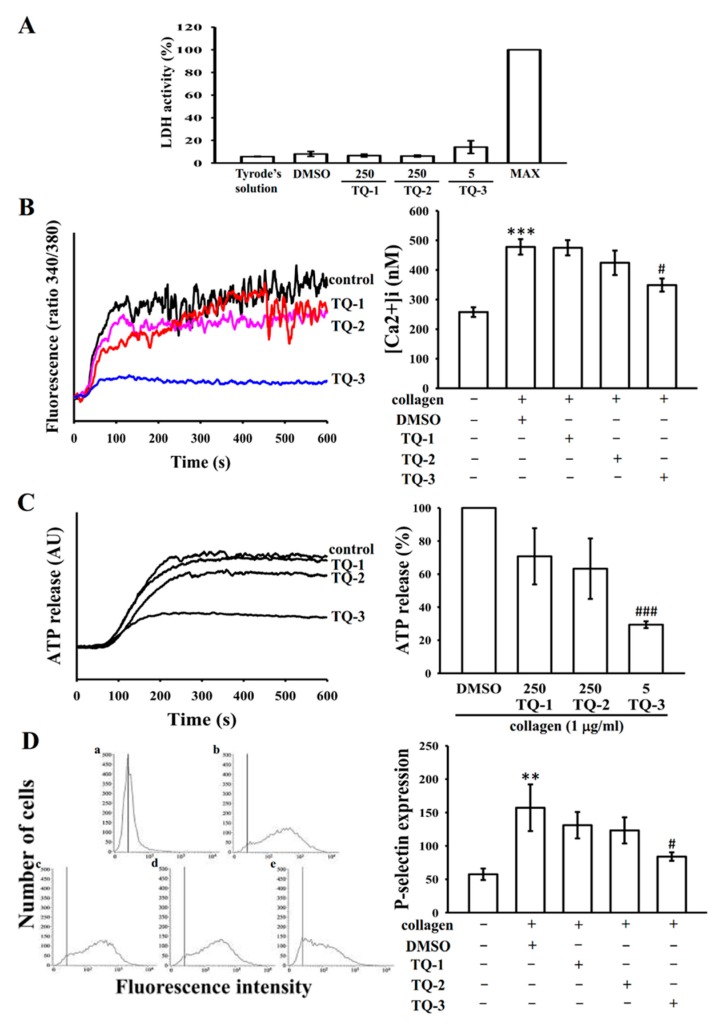
Comparative effects of TQ-1-TQ-3 on collagen induced cytotoxicity, ATP release, relative [Ca^2+^]i mobilization and on surface FITC-P-selectin expression in human platelets. Washed human platelets (3.6 × 10^8^ cells/mL) were pre-incubated with TQ-1, TQ-2 (250 μM) and TQ-3 (5 μM) or a solvent control (0.5% DMSO) to check the cytotoxicity (**A**) and subsequently treated with 1 μg/mL of collagen to stimulate ATP release reaction (**B**); to induce the cytoplasmic influx of Ca^2+^ from intracellular stores (**C**); or to check the direct binding of FITC-P-selectin (**D**) as described in the materials and methods section. Data are presented as the means ± S.E.M. (*n* = 4). ** *p* < 0.01 and *** *p* < 0.001 compared with the DMSO group. # *p* < 0.05 and ### *p* < 0.001 compared with the collagen induced group.

The effect of TQ-1, TQ-2 and TQ-3 compounds on cell toxicity was examined by measuring extracellular activity of lactate dehydrogenase (LDH). LDH is a marker of cell injury or death, which is released into the culture medium upon cell death due to the damage of plasma membrane. As shown in Figure 3A, there were no significant differences in LDH level among the three tested compounds (TQ-1, 2 and 3, vehicle (DMSO) and tyrodes solution, indicating that tested compounds do not affect platelet permeability or induce platelet cytolysis. A maximal value (MAX) of LDH was observed in the sonicated platelets, which is used as a positive control.

Stimulation of the collagen receptor GPVI leads to rapid intracellular mobilization of calcium, which is essential for platelet secretion and aggregation [19]. To investigate the effect of tested compounds on the intracellular mobilization of calcium stores, calcium levels were measured fluorometrically using the calcium-sensitive dye, Fura-2 AM. Experiments were performed in the presence of 2 mm EGTA to prevent the influx of extracellular calcium. Fura-2 AM-loaded platelets were pre-incubated with tested compounds or solvent alone for 5 min and then stimulated with collagen (1 µg/mL). Stimulation of platelets with collagen resulted in a rapid increase in intracellular calcium concentration (Figure 3B). However, this increment was significantly decreased when platelets pretreated with a maximal concentration of 5 μM TQ-3 (Figure 3B).

Intracellular [Ca^2+^] immobilization is known to be involved in the release of ATP from a dense body of platelets [20]. Because TQ-3 inhibited collagen-elevated [Ca^2+^] immobilization as shown in Figure 3B, we investigated whether this compound involves on the inhibition of ATP release. Figure 3C shows that TQ-3 significantly inhibited collagen-induced ATP release. However, collagen induced ATP release had not blocked by TQ-1 and TQ-2 even at their maximum concentration of 250 µM.

### 2.5. TQ-1, 2 and 3 on Collagen-Induced P-Selectin Expression

P-selectin is a major platelet α-granule protein that is highly expressed on the platelet surface during activation and plays important role in platelet-leukocyte and platelet-endothelial cell interactions [21]. Therefore, we examined the effects of TQ-1, 2 and 3 on collagen-induced P-selectin expression. As shown in Figure 3D, stimulation with collagen (1 µg/mL) significantly enhanced P-selectin expression in platelets, as compared to that in control untreated platelets. TQ-3 at a concentration of 5 µM significantly inhibits P-selectin expression in human washed platelets induced by collagen but TQ-1 and 2 did not effective (Figure 3D).

### 2.6. The Kinase Activities of the Src-Family Kinases Fyn and Lyn and the Tyrosine Kinase Syk are Inhibited by TQ-3

**Figure 4:**
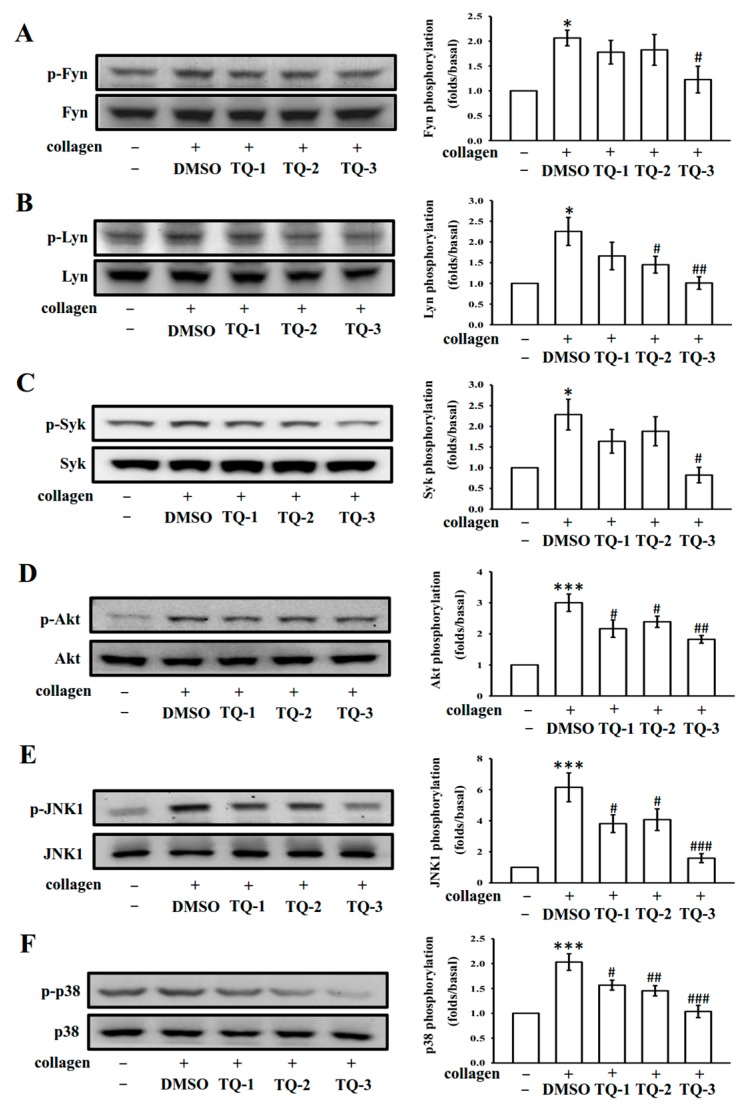
Effects of ruthenium complexes on the phosphorylation of Fyn/Lyn-Syk and MAPKs induced by collagen in human platelets. Washed platelets (1.2 × 10^9^ cells/ml) were incubated with solvent control (0.5% DMSO) or 250 μM TQ-1 and TQ-2 and 5 μM TQ-3 and then treated with 1 μg/mL collagen to induce platelet activation. The subcellular extracts were analyzed for the phosphorylation of Fyn (**A**); Lyn (**B**); Syk (**C**); Akt (**D**); JNK1(**E**) and p38 MAPK (**F**) by western blotting. Data are presented as the mean ± SEM (*n* = 4). * *p* < 0.01 and *** *p* < 0.001 compared with the DMSO group, # *p* < 0.05, ## *p* < 0.01 and ### *p* < 0.001 compared with the collagen induced group.

Binding of collagen with GPVI is reported to be mediated by the Src-family kinases Fyn and Lyn [22], resulting to activate the cytosolic tyrosine kinase Syk. In this study, these proteins were investigated to test the inhibitory effect of TQ-1, 2 and 3 on tyrosine kinase activities. To this, platelets were stimulated with collagen (1 µg/mL) in the presence and absence of TQ-1, 2 and 3 and assayed for kinase (Fyn, Lyn and Syk) phosphorylation in vitro. As shown in Figure 4A–C, the collagen induced phosphorylation of Fyn, Lyn and Syk were inhibited by TQ-3 at a lower concentration of 5 µM. However, TQ-1 and TQ-2 was not seen to inhibit the kinase phosphorylation of Fyn, Lyn and Syk even at 250 µM concentration (Figure 4A–C). The representative histograms illustrating the effect of tested compounds on SFK and Syk phosphorylation induced by collagen is shown in Figure 4, right panel.

### 2.7. TQ-3 Attenuated Collagen Stimulated Akt, JNK and p38 MAPK Phosphorylation in Platelets

Protein kinase B (Akt), JNK and p38 MAPK have been shown to play critical roles in platelet activation. To investigate whether tested ruthenium compounds inhibit these signaling events during platelet activation, we analyzed the phosphorylation of Akt, JNK and p38 MAPK. The immunoblotting analysis exhibits collagen triggered visible Akt, JNK and p38 MAPK phosphorylation in human platelets and TQ-3 more significantly (*p* < 0.01 for Akt and *p* < 0.001 for JNK and p38 than the other compounds TQ-1 and TQ-2) suppressed these phosphorylation (Figure 4D–F). These data suggest that Akt and MAPKs signaling molecules are highly implicated for the observed effects of TQ-3 against collagen-stimulated human platelets.

## 3. Discussion

Recent our studies have established that novel ruthenium based compounds TQ-5 and TQ-6 possess in vitro antiplatelet and in vivo antithrombotic effects [13,14]. These outcomes provided us with the impetus to further analyze the structure activity relationship of three (TQ-1, TQ-2 and TQ-3) newly synthesized ruthenium derived compounds in agonists induced washed human platelets. The results show that compound TQ-3 only inhibits collagen and thrombin-induced platelet aggregation but TQ-1 and TQ-2 not effective even at their maximum tested concentration of 250 µM. Moreover, TQ-3 significantly inhibited collagen-induced ATP release, Ca^2+^ mobilization and P-selectin expression but these parameters were unaltered in TQ-1 and TQ-2 treated platelets. The noted inhibitory effect of TQ-3 was attributable by inhibiting SFK-Syk-MAPKs-signaling molecules.

Platelets attach to sub-endothelial matrix protein collagen, altering their shape and leading to the release of ATP, Ca^2+^ and P-selectin. Thus, collagen acts as a platelet adhesion substrate as well as an endogenous platelet activator. Upon collagen stimulation, a rapid rise in intracellular Ca^2+^ occurs due to PI3K activation [23]. Increased Ca^2+^ consequently activates platelet granule secretion [24]. Dense granules released ATP, which causes a rapid influx of calcium by sensitizing the ionotropic receptor P2X1 [25]. In addition, P-selectin released from α granule not only aid leukocyte adhesion to endothelial cells but also acts as important for inter-platelet aggregation, stabilizing the initial GPIIb/IIIa-fibrinogen interactions, thus allowing the formation of large and stable platelet aggregates [24]. In line with these results, the effect of TQ-1, TQ-2 and TQ-3 in collagen-induced calcium mobilization, ATP secretion and P-selectin expression was tested in human platelets. The results found that among the three compounds tested, TQ-3 exerted inhibitory effects on collagen induced calcium mobilization, ATP secretion and P-selectin expression, representing that changes of these granular substances play major role in platelet activation.

Src family kinases (SFKs) are involved in the regulation of several cellular processes such as proliferation, differentiation, motility and adhesion [15]. It has been proposed that there are nine members of the Src family of tyrosine kinases, which include Src, Lck, Hck, Blk, Fyn, Lyn, Yes, Fgr and Yrk [26,27]. Previous studies described that both human and rodent platelets contain high levels of Src as well as Fyn, Lyn, Hck, Yes, Lck and Fgr [28]. Though platelets comprise elevated levels of SFKs, proposing an essential role for these enzymes in platelet function, the role of SFKs in platelet function has not been fully clarified. In platelets, SFKs, particularly Lyn and Fyn, play crucial role in downstream of collagen receptors [22]. Studies have suggested that platelets from Fyn^−/−^ mice exhibit delayed spreading on immobilized fibrinogen [29], whereas platelets from Lyn^−/−^ mice spread poorly on von Willebrand factor [30]. Studies have also proven that SFKs play a role in thromboxane generation, shape change, as well as regulation of phosphorylation of Akt [31] and ERK [32]. In this study, TQ-3 visibly reduced collagen-induced phosphorylation of Fyn, Lyn and Syk; however, TQ-1 and TQ-2 had no direct effects on these proteins, suggesting that TQ-3-mediated inhibition of platelet activation involves SFKs and Syk downstream signaling.

The Akt pathway has been discovered in platelet activation by GPVI through the regulation of the serine/threonine kinase, Akt [33]. Akt can be phosphorylated by protein kinase C and by Ca^2+^/calmodulin-dependent protein kinase independently of PI3K [34]. Activation by collagen is found to be impaired in mouse platelets deficient in Akt [35]. In this study, it has been evidenced that TQ-3 significantly inhibited collagen induced Akt phosphorylation but it did not effectively obstruct by TQ-1 and TQ-2, intending that TQ-3-potentiated inhibition of platelet activation involve the inhibition of Akt signaling pathways. Studies with inhibitors and/or genetic manipulations have demonstrated that MAPKs contribute greatly to platelet responses in various agonists [36]. Various inflammatory cytokines and stress stimuli that lead to cellular apoptosis activate JNK1/2 and p38 MAPK [37]. Previous studies have also demonstrated that JNK^−/−^ platelets are associated with increased bleeding time, decreased integrin α_IIb_β_3_ activation and severe granule secretion impairment [36]. Therefore, it seems that inhibition of the Akt, JNK and p38 phosphorylation plays important role in the platelet activation process. Consistent with this demonstration, we found that TQ-3 markedly inhibited collagen-induced Akt, JNK and p38 phosphorylation but TQ-1 and TQ-2 did not effective on these molecules.

As ruthenium is able to accomplish several oxidation states (II, III and IV) under physiological conditions and interact with a range of biomolecules, it may expedite efficient interaction of the small molecule with its molecular targets [5,38]. Besides, some ruthenium-based organometallic complexes have displayed enhanced stability in water and air [38], which may be beneficial to exert prolonged effects of the target small molecule. On studying the variation of N, N’-chelating ligands, the addition of more phenyl ring enhanced the hydrophobicity of the Ru (II) complexes. The more hydrophobic nature of the complex can be responsible for the activity toward the drug intrinsic potential. In case of the complex TQ-3 containing electron donating amino group with two phenyl group of the quinoline core could be accounted for the hydrophobicity of the complex and this might be the reason for the noted effects of TQ-3. Generally, the complexes having higher rate of hydrolysis exhibit higher cytotoxicity than those that do not hydrolyze which are inactive or weakly active. For Ru (II) arene complexes, the hydrolysis increases with the increase in electron donating ability system. A previous study performed to test the human platelet aggregation inhibitory activities of bovine, ovine and caprine kappa-casein and their tryptic hydrolysates indicated that increased hydrolysis of the compounds could enhance platelet inhibitory activities [39]. Another related study also found the comparable results that carbamoylpiperidine and nipecotoylpiperazine derivatives enhance desired antithrombotic effects with increased levels of hydrophobicity [40]. The pKa value of the quinoxaline ligand (TQ-2) is 0.6 which is lower than that of quinoline ligand (TQ-1 and TQ-3) ~ 4.9 and this value could be related to the rate of hydrolysis where the quinoline containing ligand has faster hydrolysis rate as compared to that of quinoxaline ligand. This higher pKa value of the quinoxaline ligand of TQ-3 might be accountable to its observed potent antiplatelet effects.

## 4. Methods

### 4.1. Reagents

Collagen (type I) and thrombin were purchased from Sigma (St. Louis, MO, USA). The anti-phospho-c-Jun N-terminal kinase (JNK) (Thr^183^/Tyr^185^), anti-JNK, anti-phospho-p38 mitogen-activated protein kinase (MAPK), anti-p38 MAPK and anti-phospho-Syk (Tyr^525/526^) monoclonal antibodies (mAbs) were purchased from Cell Signaling (Beverly, MA, USA). The anti-phospho-Akt (Ser^473^) and anti-Akt mAbs were purchased from Biovision (Mountain View, CA, USA). Anti-phospho-Fyn (Y^530^) mAb, anti-phospho-Lyn (Y^507^) mAb was obtained from Abcam (Cambridge, UK). The horseradish peroxidase (HRP) conjugated donkey anti-rabbit immunoglobulin G (IgG), the Hybond-P polyvinylidene difluoride (PVDF) membrane, the sheep anti-mouse IgG and the enhanced chemiluminescence western blotting detection reagent was purchased from Amersham (Buckinghamshire, UK). The TQ1, TQ-2 and TQ-3 were dissolved in DMSO and stored at 4 °C.

### 4.2. Synthesis of Ligands (L1–L3) 

All the solvents were purified according to the reported standard purification procedures. The chemicals obtained commercially were used without further purification. The starting material 5-bromo-2-nitro-benzaldehyde was prepared by following the reported procedure. Ligands 2-pyridin-2-yl-quinoline (L1) and 2-pyridin-2-yl-quinoxaline (L2) were synthesized by following the reported synthetic procedures [41]. Diphenyl-(2-pyridin-2-yl-quinolin-6-yl)-amine (L3) was prepared by adopting Friedländer quinoline synthesis followed by employing Pd-catalyzed C-N cross coupling of 6-bromo-2-pyridin-2-yl-quinoline with diphenyl amine. A representative synthetic procedure of L3 is illustrated in the following.

*Diphenyl-(2-pyridin-2-yl-quinolin-6-yl)-amine (L3).* To a flask containing 25 ml ethanolic solution of 5-bromo-2-nitro-benzaldehyde (1.15 g, 5 mM), iron powder (1.11 g, 20 mM) and 0.1N HCl (5 mL) were added and stirred vigorously at 100 °C. After 1 h, 2-acetyl pyridine (0.6 g, 5 mM) and crushed KOH (0.33 g, 6 mM) were added in portion wise and allowed to continue stirring for 2 h. Finally, the reaction mixture was cooled to RT, diluted with dichloromethane and filtered through a celite pad. The filtrate collected was then poured into water and extracted from dichloromethane. The organic layer was dried in Na_2_SO_4_, filtered and dried. The crude product was purified by column chromatography on silica gel (hexane:ethylacetate, 2:1) to obtain pale yellow solid. m.p. 118–120 °C.

*Mixture of 6-bromo-2-pyridin-2-yl-quinoline* (0.5 g, 2 mM), diphenyl amine (0.4 g, 2.4 mM), Pd_2_(dba)_3_ (0.03 g, 0.04 mM), dppf (0.06 g, 0.12 mM) and sodium *tert*-butoxide (0.3 g, 2.8 mM) were added to the freshly distilled toluene and the reaction mixture was refluxed under inert atmosphere. After 48 h, the reaction was cooled to ambient temperature; solvent was removed, washed with water and extracted from chloroform. The organic layer was dried over anhydrous Na_2_SO_4_, filtered and dried. The desired product was obtained after purification in column chromatography through silica gel in hexane:ethylacetate (1:1); ^1^H NMR (400 MHz, CDCl_3_) δ 8.64–8.63 (d, 1H, *J* = 4 Hz), 8.52–8.50 (d, 1H, *J* = 8 Hz), 8.37–8.35 (d, 1H, *J* = 8 Hz), 7.94–7.89 (t, 2H, *J* = 10 Hz), 7.78–7.75 (t, 1H, *J* = 12 Hz), 7.45–7.43 (d, 1H, *J* = 8 Hz), 7.25–7.21 (t, 6H, *J* = 8 Hz), 7.11–7.09 (d, 4H, *J* = 8 Hz), 7.03–7.00 (t, 2H, *J* = 6 Hz); ^13^C NMR (400 MHz, CDCl_3_) δ: 156.0, 153.8, 148.6, 146.9, 145.8, 144.0, 136.4, 134.9, 130.1, 129.0, 128.8, 126.6, 124.5, 123.2, 121.0, 118.7, 117.2; ESI-MS *m*/*z*: 374 (M^+^); Anal. found (calcd.) for C_26_H_19_N_3_: C, 83.58 (83.62); H, 5.20 (5.13); N, 11.35 (11.25); λ_max_, nm (CH_3_CN), 396, 347, 293, 240.

*2-Pyridin-2-yl-quinoline (L1).*^1^H NMR ( 400 MHz, CDCl_3_) δ 8.67–8.66 (d, 1H, *J* = 4 Hz), 8.61–8.59 (d, 1H, *J* = 8Hz), 8.50–8.48 (d, 1H, *J* = 8 Hz), 8.22-8.20 (d, 1H, *J* = 8 Hz), 8.14-8.12 (d, 1H, *J* = 8Hz), 7.82–7.76 (m, 2H), 7.68–7.64 (t, 1H, *J* = 8 Hz), 7.49–7.45 (t, 1H, *J* = 8 Hz), 7.30–7.27 (t, 1H, *J* = 6 Hz); ESI-MS *m*/*z*: 207.16 (M + H); Anal. found (calcd.) for C_14_H_10_N_2_: C, 81.45 (81.53); H, 4.92 (4.89); N, 13.63 (13.58); λ_max,_ nm (CH_3_CN), 335, 321, 253, 247(sh), 227.

*2-Pyridin-2-yl-quinoxaline (L2).*^1^H NMR ( 400 MHz, CDCl_3_) δ 9.90 (s, 1H), 8.73–8.72 (d, 1H, *J* = 4 Hz), 8.55–8.53 (d, 1H, *J* = 8 Hz), 8.11–8.09 (d, 2H, *J* = 8Hz), 7.86–7.82 (t, 1H, *J* = 8 Hz), 7.74–7.72 (t, 1H, *J* = 6 Hz), 7.37–7.34 (t, 1H, *J* = 6 Hz), 6.97–6.94 (d, 1H, *J* = 12 Hz); ESI-MS *m*/*z*: 208.09 (M + H); Anal. found (calcd.) for C_13_H_9_N_3_: C, 75.48 (75.35); H, 4.47 (4.38); N, 20.23 (20.28); λ_max_, nm (CH_3_CN), 335, 275, 252, 223. 

### 4.3. Synthesis of TQ1, TQ2 and TQ3 

The dimeric compound [Ru(*p*-cymene)(Cl)_2_]_2_ was synthesized following the reported procedures [42]. The complexes of TQ-1, TQ-2 and TQ-3 were prepared by following the reported methods [43]. Illustrative synthetic procedure of complexes is listed in Figure 1.

*[Ru(η^6^-cymene)(L1)Cl]BF_4_ (TQ-1).* TQ-1 was prepared by adding [Ru(*p*-cymene)(Cl)_2_]_2_ (0.1 g, 0.2 mM) dissolved in methanol into the solution of L1 (0.08 g, 0.1 mM) in methanol and stirred for 2 h at room temperature. NH_4_BF_4_ was then added and the mixture was allowed to continue stirring overnight. Finally, the solvent was removed and the solid residue obtained was dissolved in dichloromethane. Excess NH_4_BF_4_ was filtered off and the filtrate was concentrated. The solid product obtained was then wash with diethyl ether and recrystallized from chloroform and hexane yielding orange microcrystals. Yield: 82%; ^1^H NMR (400 MHz, DMSO-*d*_6_) δ 9.57–9.56 (d, 1H, *J* = 4 Hz), 8.93–8.91 (d, 1H, *J* = 8 Hz), 8.86–8.84 (d, 1H, *J* = 8 Hz), 8.76–8.74 (t, 2H, *J* = 8 Hz), 8.39–8.35 (t, 1H, *J* = 4 Hz), 8.27–8.25 (d, 1H, *J* = 8 Hz), 8.12–8.09 (t, 1H, *J* = 6 Hz), 7.96–7.93 (t, 1H, *J* = 6 Hz), 7.90–7.87 (t, 1H, *J* = 6 Hz), 6.16–6.12 (t, 2H, *J* = 8 Hz), 6.02–6.01 (d, 1H, *J* = 4 Hz), 5.96–5.94 (d, 1H, *J* = 4 Hz), 2.49 (s, 6H), 2.21 (s, 3H), 0.77–0.70 (m, 1H); ESI-MS: *m*/*z* = 477 [M^+^-BF_4_]; Anal. found (calcd.) for C_24_H_24_BClF_4_N_2_Ru: C, 51.65 (51.13); H, 4.36 (4.29); N, 5.03 (4.97). λ_max_, nm (CH_3_CN, ε × 10^2^) 421 (122), 350 (600), 335 (536), 290 (930), 265 (976), 240 (1120).

*[Ru(η^6^-cymene)(L2)Cl]BF_4_ (TQ-2).* Yield: 87%; ^1^H NMR (400 MHz, DMSO-*d*_6_) δ 10.14 (s, 1H), 9.70–9.68 (d, 1H, *J* = 8 Hz), 9.13–9.11 (d, 1H, *J* = 8 Hz), 8.76–8.75 (d, 1H, *J* = 4 Hz), 8.53–8.43 (m, 2H), 8.27–8.26 (d, 2H, *J* = 4 Hz), 8.03–8.00 (t, 1H, *J* = 6 Hz), 6.38–6.36 (d, 1H, *J* = 8 Hz), 6.32–6.30 (d, 1H, *J* = 8 Hz), 6.19–6.18 (d, 2H, *J* = 4 Hz), 2.56 (s, 6H), 2.41–2.38 (m, 1H), 2.28 (s, 3H); ESI-MS: *m*/*z* = 478 [M^+^-BF_4_]. Anal. found (calcd.) for C_23_H_23_BClF_4_N_3_Ru: C, 49.02 (48.91); H, 4.16 (4.10); N, 7.52 (7.44). λ_max_, nm (CH_3_CN, ε × 10^2^), 445 (100), 370 (527), 250 (437), 290 (452), 270 (583), 250 (704).

*[Ru(η^6^-cymene)(L3)Cl]BF_4_ (TQ-3).* Yield: 84%; ^1^H NMR (400 MHz, DMSO-*d*_6_) δ 9.49–9.48 (d, 1H, *J* = 4 Hz), 8.70–8.68 (d, 1H, *J* = 8 Hz), 8.59–8.50 (m, 3H), 8.31–8.27 (t, 1H, *J* = 8 Hz), 7.79 (s, 1H), 7.66–7.64 (d, 1H, *J* = 8 Hz), 7.47–7.28 (m, 11H), 6.11 (s, 2H), 5.97 (s, 2H), 2.18 (s, 3H), 1.12–0.99 (m, 1H), 0.76 (s, 6H); ESI-MS: *m*/*z* = 644 [M^+^-BF_4_]. Anal. found (calcd.) for C_36_H_33_BClF_4_N_3_Ru: C, 59.25 (59.15); H, 4.48 (4.55); N, 5.63 (5.75). λ_max_, nm (CH_3_CN, ε × 10^2^), 465 (187), 360 (128), 320 (354), 305 (385), 240 (316). 

### 4.4. Platelet Aggregation and ATP Release

The methods described by Sheu et al. [44] and Lin et al. [45] were followed for the preparation of human platelet suspensions. Blood was collected from healthy human volunteers who did not take medication during the preceding 2 weeks and was mixed with acid-citrate–dextrose solution (1:9). The blood samples were subjected to centrifugation at 120× *g* for 10 min and platelet-rich plasma (PRP) was collected. PRP was supplemented with PGE_1_ (0.5 μM) and heparin (6.4 IU/mL) and then incubated for 10 min at 37 °C. After centrifugation at 500× *g* for 10 min, the platelet pellets were suspended in Tyrode’s solution containing 3.5 mg mL^−1^ bovine serum albumin (BSA), pH 7.35 (NaCl 137 mM, KCl 2.7 mM, MgCl_2_ 1 mM, NaH_2_PO_4_ 0.2 mM, NaHCO_3_ 12 mM and glucose 5.5 mM). Then, PGE_1_ (0.5 μM), apyrase (1.0 U/mL) and heparin (6.4 IU/mL) were added and the mixture was incubated for 10 min at 37 °C. The mixtures were centrifuged at 500× *g* for 10 min and subjected for the repeated washing procedure. Finally, the platelet pellets were resuspended by Tyrode’s solution and then CaCl_2_ was added to platelet suspensions in which the concentration of Ca^2+^ was 1 mM. This study was approved by the Institutional Review Board of Taipei Medical University (TMU-JIRB No. 201401020) and conformed to the directives of the Helsinki Declaration.

As previously described [44,45], platelet aggregation was measured according to the turbidity of platelet suspensions and recorded by a Lumi-Aggregometer (Payton Associates, Scarborough, ON, Canada). Before the addition of collagen and thrombin to induce platelet aggregation, the platelet suspensions (3.6 × 10^8^ cells/mL) were pretreated with various concentrations (50–250 μM) of TQ1 and TQ-2 and 1–5 μM TQ-3 or an isovolumetric solvent control (0.5% DMSO) for 3 min. Light-transmission unit was used to present the extent of platelet aggregation. For the measurement of ATP release, a 20 μL of luciferin-luciferase mixture was added 1 min before adding collagen and the relative amount of ATP release was compared to the solvent control.

### 4.5. Detection of Lactate Dehydrogenase

Washed platelets (3.6 × 10^8^ cells/mL) were pretreated with TQ-1 (250 μM), TQ-2 (250 μM), TQ-3 (5 μM) or solvent control (0.1% DMSO) for 10 min at 37 °C. After centrifugation, an aliquot of the supernatant (50 µL) was measured the levels of LDH by using CytoTox 96 non-radioactive cytotoxicity assay kit from Promega (Madison, WI, USA) and the absorbance wavelength was read at 500 nm using a Synergy H1 microplate reader (BioTek, VT, USA).

### 4.6. Measurement of Relative Ca^2+^ Mobilization by Fura 2-AM Fluorescence

Citrated whole blood was centrifuged at 120× *g* for 10 min. The supernatant was incubated with Fura 2-AM (5 μM) for 1 h. Human platelets were then prepared as described above. Finally, the external Ca^2+^ concentration of the platelet suspensions was adjusted to 1 mM. The relative Ca^2+^ mobilization was measured as described previously [46].

### 4.7. Immunoblotting

Washed platelets (1.2 × 10^9^ cells/mL) were pre-incubated with 250 μM TQ-1and TQ-2 and 5 μM TQ-3 or 0.5% DMSO for 3 min, followed by the addition of collagen to trigger platelet activation. The reaction was stopped and the platelets were immediately re-suspended in 200 μL of a lysis buffer. Samples containing 80 μg of protein were separated on a 12% acrylamide gel using sodium dodecylsulfate polyacrylamide gel electrophoresis (SDS-PAGE) and the proteins were electro transferred to the PVDF membranes by using a Bio-Rad semidry transfer unit (Hercules, CA, USA). Blots were blocked with TBST (10 mM Tris-base, 100 mM NaCl and 0.01% Tween 20) containing 5% BSA for 1 h and probed with various primary antibodies. The membranes were incubated with the HRP-linked anti-mouse IgG or anti-rabbit IgG (diluted 1:3000 in TBST) for 1 h. Immunoreactive bands were detected using an enhanced chemiluminescence system. Ratios of the semi quantitative results were obtained by scanning the reactive bands and quantifying the optical density by using a video densitometer and Bio-profil Biolight software, Version V2000.01 (Vilber Lourmat, Marne-la-Vallée, France).

### 4.8. Statistical Analysis

The experimental results are expressed as the means ± SEM and are accompanied by the number of observations (n). Values of n refer to the number of experiments and each experiment was conducted using different blood donors. The paired Student's t test was used to determine significant differences in the occlusion time in mice. Differences between groups in other experiments were assessed using an analysis of variance. When this analysis indicated significant differences among group means, the groups were compared using the Student-Newman-Keuls method. *p* < 0.05 indicated statistical significance. Statistical analyses were performed using SAS Version 9.2 (SAS Inc., Cary, NC, USA).

## 5. Conclusions

This study reports that among the three compounds tested, TQ-3 has potently inhibited platelet aggregation in vitro. The inhibitory effect of TQ-3 on platelet function was attributed to the suppression of Syk-Lyn-Fyn cascade and subsequent destruction of Akt, JNK and p38 MAPKs activation. These alterations reduce the level of ATP, surface P-selectin expression and [Ca^2+]^i and ultimately inhibit platelet aggregation. The increased hydrophobicity of TQ-3 containing electron donating amino group with two phenyl group of the quinoline might be the reason for the noted effects of TQ-3. Together with the numerous advantages of organometallic complexes, the results of this study demonstrate the importance of using ruthenium-based organometallic complexes in the development of novel anti-platelet agents for the prevention and treatment of thrombotic diseases.

## Supplementary Materials

The following are available online at http://www.mdpi.com/1420-3049/23/2/477/s1, Figure S1: Absorption spectra of ligands L1–L3 in acetonitrile, Figure S2: Absorption spectra of complexes TQ1–TQ3 in acetonitrile Table S1: Crystallographic data and structure refinement parameters for TQ1–TQ3, Table S2: Selected bond lengths (Å) and angles (°) for TQ1–TQ3.

## References

[1] Davi G., Patrono C. (2007). Platelet activation and atherothrombosis. N. Engl. J. Med..

[2] Gibbins J.M. (2004). Platelet adhesion signalling and the regulation of thrombus formation. J. Cell Sci..

[3] Antithrombotic Trialists’ Collaboration (2002). Collaborative meta-analysis of randomised trials of antiplatelet therapy for prevention of death, myocardial infarction, and stroke in high risk patients. BMJ.

[4] Barrett N.E., Holbrook L., Jones S., Kaiser W.J., Moraes L.A., Rana R., Sage T., Stanley R.G., Tucker K.L., Wright B. (2008). Future innovations in anti-platelet therapies. Br. J. Pharmacol..

[5] Leung C.H., Lin S., Zhong H.J., Ma D.L. (2015). Metal complexes as potential modulators of inflammatory and autoimmune responses. Chem. Sci..

[6] Wang X., Wang X., Guo Z. (2015). Functionalization of platinum complexes for biomedical applications. Acc. Chem. Res..

[7] Feldman D.R., Bosl G.J., Sheinfeld J., Motzer R.J. (2008). Medical treatment of advanced testicular cancer. JAMA.

[8] Dorcier A., Ang W.H., Bolan˜o S., Gonsalvi L., Jeannerat L.J., Laurenczy G., Peruzzini M., Phillips A.D., Zanobini F., Dyson P.J. (2006). In Vitro Evaluation of rhodium and osmium RAPTA analogues: The case for organometallic anticancer drugs not based on ruthenium. Organometallics.

[9] Bergamo A., Sava G. (2011). Ruthenium anticancer compounds: Myths and realities of the emerging metal-based drugs. Dalton Trans..

[10] Lentz F., Drescher A., Lindauer A., Henke M., Hilger R.A., Hartinger C.G., Scheulen M.E., Dittrich C., Keppler B.K., Jaehde U. (2009). Central European Society for Anticancer Drug Research-EWIV. Pharmacokinetics of a novel anticancer ruthenium complex (KP1019, FFC14A) in a phase I dose-escalation study. Anticancer Drugs.

[11] Scolaro C., Bergamo A., Brescacin L., Delfino R., Cocchietto M., Laurenczy G., Geldbach T.J., Sava G., Dyson P.J. (2005). In vitro and in vivo evaluation of ruthenium(II)-arene PTA c complexes. J. Med. Chem..

[12] Strohfeldt K.A. (2015). Essentials of Inorganic Chemistry.

[13] Khamrang T., Hung K.C., Hsia C.H., Hsieh C.Y., Velusamy M., Jayakumar T., Sheu J.R. (2017). Antiplatelet activity of a newly synthesized novel ruthenium (II): A potential role for Akt/JNK signaling. Int. J. Mol. Sci..

[14] Hsia C.H., Velusamy M., Sheu J.R., Khamrang T., Jayakumar T., Lu W.J., Lin K.H., Chang C.C. (2017). A novel ruthenium (II)-derived organometallic compound, TQ-6, potently inhibits platelet aggregation: Ex vivo and in vivo studies. Sci. Rep..

[15] Morris R.E., Aird R.E., Murdoch P.S., Chen H., Cummings J., Hughes N.D., Parsons S., Parkin A., Boyd G., Jodrell D.I., Sadler P.J. (2001). Inhibition of cancer cell growth by ruthenium(II) arene complexes. J. Med. Chem..

[16] Türkmen H., Kani I., Çetinkaya B. (2012). Transfer Hydrogenation of Aryl Ketones with Half-Sandwich Ru(II) Complexes That Contain Chelating Diamines. Eur. J. Inorg. Chem..

[17] Muthuramalingam S., Khamrang T., Velusamy M., Mayilmurugan M. (2017). Catalytic fixation of atmospheric carbon dioxide by copper(II) complexes of bidentate ligands. Dalton Trans..

[18] Tsolis T., Manos M.J., Karkabounas S., Zelovitis I., Garoufis A. (2014). Synthesis, X-ray structure determination, cytotoxicity and interactions with 9-methylguanine, of ruthenium(II) η6-arene complexes. J. Organomet. Chem..

[19] Smith J.B., Selak M.A., Dangelmaier C., Daniei J.L. (1992). Cytosolic calcium as a second messenger for collagen-induced platelet responses. Biochem. J..

[20] Charo I.F., Feinman R.D., Detwiler T.C. (1976). Inhibition of platelet secretion by an antagonist of intracellular calcium. Biochem. Biophys. Res. Commun..

[21] von Hundelshausen P., Weber C. (2007). Platelets as immune cells: Bridging inflammation and cardiovascular disease. Circ. Res..

[22] Quek L.S., Pasquet J.M., Hers I., Cornall R., Knight G., Barnes M., Hibbs M.L., Dunn A.R., Lowell C.A., Watson S.P. (2000). Fyn and Lyn phosphorylate the Fc receptor gamma chain downstream of glycoprotein VI in murine platelets, and Lyn regulates a novel feedback pathway. Blood.

[23] Guidetti G.F., Lova P., Bernardi B., Campus F., Baldanzi G., Graziani A., Balduini C., Torti M. (2008). The Gi-coupled P2Y12 receptor regulates diacylglycerol-mediated signaling in human platelets. J. Biol. Chem..

[24] Flaumenhaft R. (2003). Molecular basis of platelet granule secretion. Arterioscler. Thromb. Vasc. Biol..

[25] Mahaut-Smith M.P., Ennion S.J., Rolf M.G., Evans R.J. (2000). ADP is not an agonist at P2X(1) receptors: Evidence for separate receptors stimulated by ATP and ADP on human platelets. Br. J. Pharmacol..

[26] Thomas S.M., Brugge J.S. (1997). Cellular functions regulated by Src family kinases. Annu. Rev. Cell Dev. Biol..

[27] Boggon T.J., Eck M.J. (2004). Structure and regulation of Src family kinases. Oncogene.

[28] Stenberg P.E., Pestina T.I., Barrie R.J., Jackson C.W. (1997). The Src family kinases, Fgr, Fyn, Lck, and Lyn, colocalize with coated membranes in platelets. Blood.

[29] Reddy K.B., Smith D.M., Plow E.F. (2008). Analysis of Fyn function in hemostasis and alphaIIbbeta3-integrin signaling. J. Cell Sci..

[30] Yin H., Liu J., Li Z., Berndt M.C., Lowell C.A., Du X. (2008). Src family tyrosine kinase Lyn mediates VWF/GPIb-IX-induced platelet activation via the cGMP signaling pathway. Blood.

[31] Kim S., Jin J., Kunapuli S.P. (2006). Relative contribution of G-protein-coupled pathways to protease-activated receptor-mediated Akt phosphorylation in platelets. Blood.

[32] Garcia A., Shankar H., Murugappan S., Kim S., Kunapuli S.P. (2007). Regulation and functional consequences of ADP receptor-mediated ERK2 activation in platelets. Biochem. J..

[33] Jackson S.P., Yap C.L., Anderson K.E. (2004). Phosphoinositide 3-kinases and the regulation of platelet function. Biochem. Soc. Trans..

[34] Deb T.B., Coticchia C.M., Dickson R.B. (2004). Calmodulin-mediated activation of Akt regulates survival of c-Myc-overexpressing mouse mammary carcinoma cells. J. Biol. Chem..

[35] Chen J., De S., Damron D.S., Chen W.S., Hay N., Byzova T.V. (2004). Impaired platelet responses to thrombin and collagen in AKT-1-deficient mice. Blood.

[36] Adam F., Kauskot A., Nurden P., Sulpice E., Hoylaerts M.F., Davis R.J., Rosa J.P., Bryckaert M. (2010). Platelet JNK1 is involved in secretion and thrombus formation. Blood.

[37] Chang L., Karin M. (2001). Mammalian MAP kinase signalling cascades. Nature.

[38] Page S. (2012). Ruthenium compounds as anticancer agents. Educ. Chem..

[39] Manso A., Escudero C., Alijo M., López-Fandiño R. (2002). Platelet aggregation inhibitory activity of bovine, ovine, and caprine kappa-casein macropeptides and their tryptic hydrolysates. J. Food Prot..

[40] Alevriadou B.R., McIntire L.V., Lasslo A. (1992). Inhibition of platelet adhesion and thrombus formation on a collagen-coated surface by novel carbamoylpiperidine antiplatelet agents. Biochim. Biophys. Acta.–Mol. Cell Res..

[41] Li A.H., Ahmed E., Chen X., Cox M., Crew A.P., Dong H.O., Jin M., Ma L., Panicker B., Siu K.W. (2007). A highly effective one-pot synthesis of quinolines from o-nitroarylcarbaldehydes. Org. Biomol. Chem..

[42] Bennelt M.A., Smith A.K. (1974). Arene ruthenium(II) complexes formed by dehydrogenation of cyclohexadienes with ruthenium(III) trichloride. J. Chem. Soc. Dalton Trans..

[43] Bugarcic T., Habtemariam A., Deeth R.J., Fabbiani F.P.A., Parsons S., Sadler P.J. (2009). Ruthenium(II) arene anticancer complexes with redox-active diamine ligands. Inorg. Chem..

[44] Sheu J.R., Lee C.R., Lin C.H., Hsiao G., Ko W.C., Chen Y.C., Yen M.H. (2000). Mechanisms involved in the antiplatelet activity of Staphylococcus aureus lipoteichoic acid in human platelets. Thromb. Haemost..

[45] Lin K.H., Kuo J.R., Lu W.J., Chung C.L., Chou D.S., Huang S.Y., Lee H.C., Sheu J.R. (2013). Hinokitiol inhibits platelet activation ex vivo and thrombus formation in vivo. Biochem. Pharmacol..

[46] Hsiao G., Lin K.H., Chang Y., Chen T.L., Tzu N.H., Chou D.S., Sheu J.R. (2005). Protective mechanisms of inosine in platelet activation and cerebral ischemic damage. Arterioscler. Thromb. Vasc. Biol..

